# Getting valuable and valid insight in life after ICU: evaluating the representativeness of a large cohort of ICU survivors

**DOI:** 10.1093/intqhc/mzaf078

**Published:** 2025-08-12

**Authors:** Julian van Gemert, Marieke Zegers, Eddy Adang, Ferishta Bakhshi-Raiez, Koen Simons, Stijn Corsten, Brigitte Westerhof, Esther Ewalds, Marijke de Vries, Dave Dongelmans, Nicolette de Keizer, Mark van den Boogaard

**Affiliations:** Department of Intensive Care Medicine, Radboud University Medical Center, Geert Grooteplein 10, Nijmegen, Gelderland, 6500 HB, The Netherlands; Department of Intensive Care Medicine, Radboud University Medical Center, Geert Grooteplein 10, Nijmegen, Gelderland, 6500 HB, The Netherlands; Department of Health Evidence, Radboud University Medical Center, Geert Grooteplein 10, Nijmegen, Gelderland, 6500 HB, The Netherlands; Department of Medical Informatics, Amsterdam University Medical Center, Meibergdreef 9, Amsterdam, Noord-Holland, 1105 AZ, The Netherlands; National Intensive Care Evaluation (NICE) Foundation, Postbus 23640, Amsterdam, Noord-Holland, 1100 EC, The Netherlands; Department of Intensive Care Medicine, Jeroen Bosch Hospital, Henri Dunantstraat 1, ‘s-Hertogenbosch, Noord-Brabant, 5223 GZ, The Netherlands; Department of Intensive Care Medicine, Canisius Wilhelmina Hospital, Jonkerbos 100, Nijmegen, Gelderland, 6532 SZ, The Netherlands; Department of Intensive Care Medicine, Rijnstate Hospital, Wagnerlaan 55, Arnhem, Gelderland, 6815 AD, The Netherlands; Department of Intensive Care Medicine, Bernhoven Hospital, Nistelrodeseweg 10, Uden, Noord-Brabant, 5406 PT, The Netherlands; Department of Intensive Care Medicine, Maasziekenhuis Pantein, Dokter Kopstraat 1, Beugen, Noord-Brabant, 5835 DV, The Netherlands; Department of Medical Informatics, Amsterdam University Medical Center, Meibergdreef 9, Amsterdam, Noord-Holland, 1105 AZ, The Netherlands; National Intensive Care Evaluation (NICE) Foundation, Postbus 23640, Amsterdam, Noord-Holland, 1100 EC, The Netherlands; Department of Medical Informatics, Amsterdam University Medical Center, Meibergdreef 9, Amsterdam, Noord-Holland, 1105 AZ, The Netherlands; National Intensive Care Evaluation (NICE) Foundation, Postbus 23640, Amsterdam, Noord-Holland, 1100 EC, The Netherlands; Quality of Care and Digital Health, Amsterdam Public Health, Meibergdreef 9, Amsterdam, Noord-Holland, 1105 AZ, The Netherlands; Department of Intensive Care Medicine, Radboud University Medical Center, Geert Grooteplein 10, Nijmegen, Gelderland, 6500 HB, The Netherlands

**Keywords:** representativeness, ICU survivors, critical care, patient-reported outcomes, bias

## Abstract

**Background:**

Measuring patient-reported outcome measures (PROMs) is essential for improving intensive care medicine, but it is challenging and prone to bias. This study investigates the representativeness of a multicenter, PROM-based cohort of intensive care unit (ICU) survivors.

**Methods:**

Demographic, clinical, and ICU admission characteristics of the multicenter, PROM-based cohort (*n* = 6 ICUs) were compared to those of all ICU survivors in Dutch hospitals (*n* = 73 ICUs), in the years 2019 and 2022, based on data from the National Intensive Care Evaluation registry.

**Results:**

Comparison between the PROM-based cohort (*n* = 2454) and the national registry cohort (*n* = 89 154) revealed predominantly similarities in demographic, clinical, and ICU admission characteristics. Nevertheless, ICU survivors in the PROM-based cohort had a higher severity of illness (59 vs. 56 points) and mortality probability (19% vs. 16%), were more often mechanically ventilated during the first 24 h after ICU admission (49% vs. 34%), had higher ICU and hospital lengths of stay (respectively, 4.7 vs. 3.6 days, and 16 vs. 14 days), and lower mortality rates in-hospital and at 3, 6, and 12 months after ICU admission (respectively, 1.4% vs. 4.6%, 4.2% vs. 10%, 6.2% vs. 13%, and 9.2% vs. 17%).

**Conclusion:**

ICU survivors in the PROM-based cohort share similar demographic, clinical, and ICU admission characteristics with the national ICU population. However, severity of illness, lengths of stay, and mortality rates deviate from the national registry cohort. These findings highlight external validity concerns, urging researchers and policymakers to consider this when using outcome data from a PROM-based cohort.

## Introduction

Advances in critical care medicine over recent decades have resulted in more intensive care unit (ICU) survivors [1]. However, survival is often linked to persistent complications, including long-term morbidity, ICU-acquired weakness, frailty, disability, and mental health issues like Post Traumatic Stress Disorder (PTSD), depression, and anxiety [2]. Consequently, long-term functional outcomes and quality of life (QoL) measures are increasingly recognized as crucial in clinical research [3–5]. These patient-reported outcome measures (PROMs), commonly assessed through questionnaires, are used to guide care, aiming to reduce symptoms and improve QoL [6].

For this purpose, a PROM-based, multicenter cohort was initiated in 2016 in the Netherlands, called MONITOR-IC (www.monitor-ic.nl). Within this ongoing cohort study, short- and long-term PROMs on physical, mental, cognitive, and QoL domains are being collected up to 5 years after ICU admission [7]. All ICU admissions in participating hospitals are approached for participation. Data are being collected in a dynamic database that currently encompasses over 15 000 ICU patients, representing a heterogeneous cohort. This extensive database of PROMs has repeatedly been used for, among other purposes, developing prediction models to support the decision-making process by ICU clinicians, and to improve care. The PROSPECT model has been developed to predict the post-intensive care syndrome, including short- and long-term, physical (e.g. fatigue and frailty), mental (e.g. anxiety, depression, and PTSD), and cognitive (e.g. memory and attention) post-ICU health problems [8]. In particular, this has led to novel insights regarding the long-term PROMs of ICU survivors infected with the corona virus [9, 10]. Moreover, the PREPARE model has been developed to predict changes in QoL of ICU survivors [11–13]. These models help physicians prepare patients and relatives for life after ICU. Within these models, pre-ICU QoL and health status have been identified as crucial predictors for both short- and long-term outcomes [14].

Measuring patient-reported outcomes is fundamental for improving intensive care medicine but remains challenging. Despite efforts to include all ICU patients, some do not participate due to vulnerability or unwillingness [15–18]. Hence, before widely implementing prediction models and other insights based on PROM-based cohorts, the representativeness of such a cohort needs to be validated. The Dutch quality registry, i.e. the National Intensive Care Evaluation (NICE) registry is used for this validation [19]. This registry includes all Dutch ICU admissions with clinical, demographic, and ICU characteristics, but no PROM data. The current study aims to investigate whether demographic, clinical, and ICU admission characteristics of ICU survivors, who consented and submitted PROM-data, differ from those of all ICU survivors in the Netherlands, both in the general ICU population and within specific subgroups.

## Methods

### Study design

The current study is a cross-sectional study comparing a subpopulation of ICU survivors who gave informed consent for ICU follow-up and submitted PROM-data, to all ICU survivors in Dutch hospitals (excluding the subpopulation), registered in the NICE registry [19]. The PROM-based cohort is called the MONITOR-IC (ClinicalTrials.gov: NCT03246334) and its protocol has previously been published and can be referred to for further details [7]. The study has been approved by the local ethics committee of the Radboud University Medical Center, Committee on Research Involving Human Subjects, region Arnhem-Nijmegen, The Netherlands (2016-2724), and is conducted in accordance with the declaration of Helsinki [7].

### Study population

In the MONITOR-IC, ICU patients were eligible to participate when they (i) were aged 16 years or older, (ii) were admitted to the ICU for 12 h or longer, (iii) had a life expectancy of more than 48 h, (iv) did not receive palliative care, and (v) were able to read or speak the Dutch language. All included patients, or their legal representatives, provided written informed consent.

In the present study, MONITOR-IC patients were included when they (i) survived ICU stay, (ii) were admitted to the ICU in the year 2019 or 2022 [to avoid bias due to the COVID-19 (coronavirus disease 2019) pandemic], and (iii) were admitted to one of the six MONITOR-IC hospitals: Radboud University Medical Center, Canisius Wilhelmina Hospital, Bernhoven Hospital, Maasziekenhuis Pantein, Jeroen Bosch Hospital, and Rijnstate Hospital (one academic, three teaching, and two general hospitals; including one cardiothoracic center).

The national registry cohort was corrected for the inclusion criteria of the MONITOR-IC and the current study. ICU patients within this cohort were included when they (i) were aged 16 years or older, (ii) were admitted to the ICU for 12 h or longer, (iii) survived ICU stay, and (iv) were admitted to the ICU in the year 2019 or 2022. The NICE registry lacks data on life expectancy at ICU admission, palliative care indication, or the literacy in Dutch language. In case a patient was admitted multiple times to the ICU within one hospital admission, only the index ICU admission was included. This ensured cohort homogeneity, as MONITOR-IC does not allow multiple inclusions per patient.

### Data collection

For the purpose of this study, data of all ICU patients in the Netherlands, recorded in the NICE registry, were used. The data include demographic, clinical, and ICU admission data collected in the first 24 h after ICU admission, as well as ICU and hospital mortality and length of stay (LOS), but did not include PROM data [19]. Mortality after hospital discharge was retrieved through the Vektis database, a Dutch administrative database of all health insurance companies on medical insurance claims [20]. Within this database, the PROM-based cohort could be retrieved (and distinguished from the national registry cohort) using a unique, and random, NICE identifier (available in the MONITOR-IC and NICE datasets).

### Study variables

Demographic, clinical, and ICU admission characteristics of ICU survivors were used to study the representativeness of the study population. Demographic data such as age, gender, height, and weight were included. Additionally, data on clinical and ICU admission characteristics were reported, encompassing preexisting comorbidities, admission type, admission source, planned admission, and hospital type. Moreover, the severity of illness score [Acute Physiology and Chronic Health Evaluation (APACHE) III score], APACHE IV mortality probability, mechanical ventilation within the first 24 h of ICU admission, cardiothoracic surgery, primary admission diagnosis (such as community-acquired pneumonia, sepsis, out-of-hospital cardiac arrest, trauma, or stroke), ICU LOS, hospital LOS, hospital mortality, and mortality rates at 3, 6, and 12 months post-ICU admission were studied [21]. Utilizing these data, additional variables were determined or computed, including body mass index (BMI), and APACHE IV risk categories [based on the APACHE IV mortality probability: high (≥70%), medium (≥30% to <70%), and low (<30%)]. These thresholds are routinely applied in Dutch ICU benchmarking practice by the NICE registry, and are based on tertiles of the absolute number of nonsurvivors [22].

### Statistical analyses

To study the representativeness of the PROM-based cohort, descriptive statistics assessed the differences in demographic, clinical, and ICU admission characteristics between the PROM-based cohort and the national registry cohort. Cardiothoracic surgery patients were separately analyzed and excluded from the main analyses, due to the known overrepresentation of these patients in the PROM-based cohort and the inability to generalize these patients to the general ICU population [12]. Additionally, the PROM-based cohort was compared with all ICU survivors in the six MONITOR-IC hospitals (excluding the PROM-based cohort) as a sensitivity analysis. Moreover, academic and nonacademic hospital patients were separately analyzed.

This study included all Dutch ICU patients. In studies with full coverage or large sample sizes, standardized mean difference (SMD) is used to assess differences between cohorts [23]. SMD is calculated using the formula:  (M1-M2)/SDpooled, where *M*_1_ and *M*_2_ are the means of the two groups and SD_pooled_ is the standard deviation of the pooled groups. A biostatistician was consulted for the selection of the SMD as an appropriate method for assessing differences between the PROM-based cohort and the national registry cohort. An SMD > 0.10 was considered as a clinically relevant imbalance in characteristics between the two groups [24, 25]. Data were presented as means with SDs for continuous variables (Central Limit Theorem), and counts with percentages (%) for categorical variables [26]. All analyses were performed with R software, version 4.2.1 (R Foundation for Statistical Computing, Vienna, Austria), using the following packages: haven, dplyr, lubridate, gtsummary, and gt. Supplementary tables were added on rates of missing data, and additional (reference) comparisons.

## Results

### Study participants

**Figure 1:**
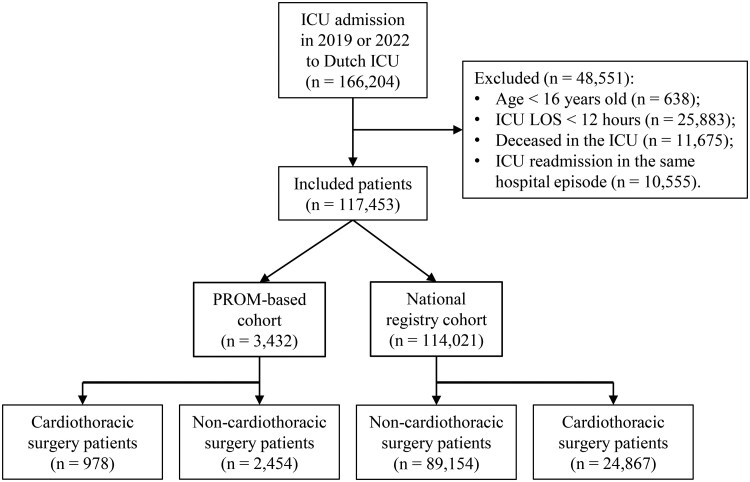
Flow diagram illustrating the selection process of ICU survivors (from 2019 to 2022)

The analysis of two main groups was performed: noncardiothoracic surgery ICU survivors within the PROM-based study (*n* = 2454) versus noncardiothoracic surgery ICU survivors within the national registry cohort (*n* = 89 154). The selection process is visually described in Fig. 1. In the years 2019 and 2022, a total of 166 204 patients were admitted to ICUs across the 73 hospitals in the Netherlands (9 academic, 23 teaching, and 41 general hospitals; including 13 cardiothoracic centers). Out of these admissions, 117 453 patients (70.7%) were included in the current study. The exclusion criteria were younger than 16 years old (0.4%), ICU LOS shorter than 12 h (15.6%), deceased in the ICU (7.0%), and ICU readmission within the same hospital episode (6.4%). Among the included patients, 3432 patients (2.9%) participated in the PROM-based study. Cardiothoracic surgery patients were separately analyzed (28.5% of the PROM-based cohort, and 21.8% of the national registry cohort).

Within the PROM-based cohort and the national registry cohort, missing data of at least 1% were observed to be present for the following variables: BMI (7.2% and 3.6%, respectively), admission type (national registry cohort: 1.4%), APACHE III score, and APACHE IV mortality probability and underlying risk categories (for all; national registry cohort: 1.3%) (Supplementary Table 1). Given the low numbers of missings, no imputation was performed.

### Comparison with national registry cohort

Compared to all ICU survivors in the national registry cohort, the PROM-based cohort exhibited no differences in age, gender, BMI, or the prevalence of patients with at least one comorbidity (Table 1). With respect to clinical and ICU admission characteristics, the admissions of ICU survivors in the PROM-based cohort were less frequently medical admissions (60% vs. 63%) and more often emergency surgical admissions (17% vs. 13%). In contrast, the admission source and the number of planned admissions were not different between cohorts. The PROM-based cohort consisted of relatively more admissions in academic and teaching hospitals, and less admissions in general hospitals. Moreover, the PROM-based cohort displayed a higher mean APACHE III score (59 vs. 56) and APACHE IV mortality probability (0.19 vs. 0.16), while the distribution across APACHE IV risk groups was not different between cohorts. Mechanical ventilation within the first 24 h after ICU admission was more frequently applied in the PROM-based cohort (49% vs. 34%). Furthermore, the incidences of the primary admission diagnoses did not clinically differ between cohorts. Among the ICU survivors in the PROM-based cohort, mean ICU LOS (4.7 vs. 3.6 days) and hospital LOS (16 vs. 14 days) were longer, and lower mortality rates in the hospital (1.4% vs. 4.6%), at 3 (4.2% vs. 10%), 6 (6.2% vs. 13%), and 12 months (9.2% vs. 17%) were observed.

**Table 1.** Differences in demographic, clinical, and ICU admission characteristics between ICU survivors in the PROM-based cohort and in the national registry cohort, admitted in 2019 or 2022

Variable	**PROM-based cohort**, *N* = 2454^a^	**National registry cohort**, *N* = 89 154^a^	SMD
Age (years)	62 (±15)	61 (±17)	0.03
Gender			0.04
Female	984 (40%)	37 539 (42%)	
Male	1469 (60%)	51 612 (58%)	
Body mass index (kg/m²)	27.1 (±5.7)	26.9 (±6.2)	0.03
At least one comorbidity^b^	710 (29%)	22 817 (26%)	0.08
Admission type			0.11
Medical	1474 (60%)	55 721 (63%)	
Emergency surgical	404 (16%)	11 247 (13%)	
Planned surgical	576 (23%)	21 707 (24%)	
Admission source			0.07
Operating room	781 (32%)	28 182 (32%)	
Emergency room	843 (34%)	31 311 (35%)	
Nursing ward	595 (24%)	19 956 (22%)	
ICU/CCU/Rec/Spec/MCU^c^	52 (2.1%)	1562 (1.8%)	
Other	183 (7.5%)	7769 (8.8%)	
Planned admission	524 (21%)	18 873 (21%)	0.00
Hospital type			0.77
Academic	970 (40%)	17 523 (20%)	
Teaching	1263 (51%)	37 632 (42%)	
General	221 (9.0%)	33 999 (38%)	
APACHE III score	59 (±25)	56 (±25)	0.15
APACHE IV			
Mortality probability	0.19 (±0.20)	0.16 (±0.19)	0.13
Risk category			0.08
Low	1949 (80%)	72 384 (83%)	
Medium	370 (15%)	12 435 (14%)	
High	113 (4.6%)	2809 (3.2%)	
Mechanical ventilation (first 24 h)	1204 (49%)	30 630 (34%)	0.30
Primary admission diagnosis			
Community acquired pneumonia	178 (7.3%)	5205 (5.8%)	0.06
Sepsis	209 (8.5%)	6434 (7.2%)	0.05
Out-of-hospital cardiac arrest	125 (5.1%)	2729 (3.1%)	0.10
Trauma	203 (8.3%)	6541 (7.3%)	0.03
Stroke	50 (2.0%)	3001 (3.4%)	0.08
LOS (days)			
ICU	4.7 (±7.8)	3.6 (±7.1)	0.15
Hospital	16 (±16)	14 (±16)	0.12
Mortality			
Hospital	35 (1.4%)	4136 (4.6%)	0.19
3 months	103 (4.2%)	9039 (10%)	0.23
6 months	153 (6.2%)	11 577 (13%)	0.23
12 months	226 (9.2%)	15 243 (17%)	0.23

a Mean (±SD) or frequency (%).

b Immunological insufficiency, renal insufficiency, metastasized neoplasm, respiratory insufficiency, cardiovascular insufficiency, hematological malignancy, or liver cirrhosis.

c Intensive care unit, coronary care unit, recovery, special care unit, or medium care unit.

### Subgroup analyses

The comparison on the cardiothoracic surgery patients between the ICU survivors in the PROM-based cohort and the ICU survivors in the national registry cohort is included in Supplementary Table 2. Within this subgroup analysis, less clinically relevant differences were observed between cohorts, when compared to noncardiothoracic surgery patients. The LOSs were not different between cohorts, neither were the mortality rates in the hospital, and at 3 and 6 months after ICU admission.

Additionally, the comparison between the ICU survivors in the PROM-based cohort and ICU survivors not participating in the PROM-based study within the six MONITOR-IC hospitals is included in Supplementary Table 3.

The comparison on the academic and nonacademic ICU patients separately between the ICU survivors in the PROM-based cohort and the ICU survivors in the national registry cohort is included in Supplementary Tables 4 and 5. In these subgroup analyses, it is observed that academic ICU patients in the PROM-based cohort originate more often from the operating room and their admission reason is more often planned (surgical), when compared to the academic ICU patients in the national registry cohort. Furthermore, their APACHE III score, APACHE IV mortality probability, and risk group profile were lower. In contrast, the nonacademic ICU patients in the PROM-based cohort originate less often from the operating room and their admission reason is less often planned (surgical), when compared to the nonacademic ICU patients in the national registry cohort. Moreover, their APACHE III score, APACHE IV mortality probability and risk group profile were higher, and they were admitted to the ICU and the hospital for longer. The other characteristics, such as mortality rates, of these patient groups were comparable to the main comparison of the noncardiothoracic surgery patients.

## Discussion

### Statement of principal findings

In this study, the representativeness of ICU survivors in a large, PROM-based cohort was evaluated. Most of the demographic, clinical, and ICU admission characteristics of the PROM-based cohort align closely with those of ICU survivors in the national registry cohort, suggesting that the PROM-based cohort is representative of the broader population of ICU survivors. However, the PROM-based cohort of ICU survivors was more severely ill, as expressed by higher APACHE scores, exhibited longer ICU LOS and hospital LOS, and had lower mortality rates, when compared to the rest of the ICU survivors in Dutch hospitals. However, when comparing more homogenous groups, such as the cardiothoracic surgery patients, more similarities, in terms of demographics and clinical and admission characteristics, with the national registry cohort were observed [13, 27].

### Interpretation within the context of the wider literature

The observed higher APACHE III scores and APACHE IV mortality probabilities, the increased rates of mechanically ventilated patients (within the first 24 h after ICU admission), and the longer ICU and hospital LOS among the ICU survivors in the PROM-based cohort suggest a more severely impaired group of ICU survivors. Paradoxically, long-term mortality rates were lower in this cohort. These findings suggest that results from this survivor cohort should be approached with caution, as outcomes can be overestimated due to the more severely ill patient group included and longer LOS, or, on the other hand, underestimated due to the lower mortality rates.

When stratifying both cohorts based on hospital type, academic ICU patients in the PROM-based cohort had lower APACHE III scores, while non-academic ICU patients had higher APACHE III scores compared to the national registry cohort. The rates of planned (surgical) admissions in both cohorts likely explain their respective APACHE III scores. Furthermore, the higher incidence of mechanically ventilated patients in the PROM-based cohort may be attributed to the larger proportion of ICU survivors admitted to academic hospitals. In academic hospitals, beds without mechanical ventilation are typically situated in intermediate care units, whereas in non-academic hospitals, such beds are most often found within the ICU [28]. Inevitably, prospective large cohort studies are prone to selective participation, commonly known as selection bias [17]. Specifically, vulnerable ICU survivors who were severely ill at admission of those with a short LOS may have limited willingness to fill in questionnaires and participate in PROM-based research [15–18]. This likely explains the observed longer LOSs and lower mortality rates in the PROM-based cohort.

### Strengths and limitations

This PROM-based cohort study is unique in both its approach and scale, as it reaches out to all eligible patients admitted to the ICU across participating hospitals, and it currently encompasses patient-reported outcome data of a heterogeneous population of more than 15 000 ICU survivors. This approach gives us the most comprehensive sample of ICU survivors, while taking into account patients’ consent. Moreover, the current study is globally the first study evaluating the representativeness of such a large PROM-based ICU cohort. Furthermore, through the use of the NICE registry, having a national coverage, and complete and comprehensive data about demographic, clinical, and ICU admission characteristics were available for the PROM-based cohort as well as for the broader population of ICU survivors. All of this allows an accurate evaluation of the representativeness of the PROM-based cohort.

This study assesses the representativeness of a PROM cohort through demographic and clinical characteristics. However, these characteristics may not fully capture all dimensions that influence PROM outcomes, such as personality traits (e.g. resilience, optimism, insight, coping style), and socioeconomic factors [29, 30]. Furthermore, selection bias likely affects the PROM-based cohort, as severely ill or vulnerable survivors may be underrepresented due to lower participation rates, limiting the generalizability of PROM outcomes. Not all inclusion criteria that were applied to the PROM-based cohort could be applied to the national registry cohort. It was not possible to exclude patients having a life expectancy of less than 48 h at the time of ICU admission, patients who received palliative care, and patients who were not able to read or speak the Dutch language, from the national registry cohort. This could have possibly led to higher mortality rates in the national registry cohort. Moreover, only readmissions during the same hospital admission could be excluded in the national registry cohort, as patients who were readmitted to the ICU after hospital discharge were not identifiable. Thus, duplicate patients in the national registry cohort could not completely be avoided. Additionally, some ICU survivors in the non-PROM group may have received ICU aftercare including PROM collection, but the NICE registry lacks data on such follow-up care outside the MONITOR-IC cohort, limiting comparisons between groups. Lastly, the data analysis within this study was limited to mostly (clinical) data registered in the first 24 h of ICU admission.

## Conclusions and implications for policy, practice, and research

This study demonstrates that ICU survivors willing to participate in a PROM-based cohort exhibit largely similar demographic, clinical, and admission characteristics compared to the national ICU survivor population. However, notable deviations in severity of illness, LOSs, and mortality rates between the PROM-based cohort and the national cohort reflect selection bias, and highlight concerns about the external validity of PROM-based research. Researchers and policy makers should take these differences into account when using a PROM-based cohort e.g. for developing and implementing prediction models, to support treatment decisions in the overall ICU population.

## Supplementary Material

mzaf078_Supplementary_Data

## Data Availability

The datasets used in this study are not publicly available due to the data sharing agreements with the participating hospitals. Access to the data might only be provided after explicit consent from each of the participating hospitals and under strict conditions, as described (in Dutch) at https://www.stichting-nice.nl/extractieverzoeken.jsp. The (programming) code used during the data analysis of the current study is available from the corresponding author.
