# Supplementary material for: Getting valuable and valid insight in life after ICU: evaluating the representativeness of a large cohort of ICU survivors
Source: Int J Qual Health Care. 2025 Aug 12;37(3):mzaf078. doi: 10.1093/intqhc/mzaf078 (PMC12459092; doi:10.1093/intqhc/mzaf078)
Supplement: mzaf078_Supplementary_Data [file mzaf078_supplementary_data.zip › Supplementary_material.docx]

Supplemental Table 1. Rates of missing data in the PROM-based cohort and the national registry cohort of ICU survivors, admitted in 2019 or 2022.

| **Variable** | **PROM-based cohort**,  N = 3,432*^1^* | **National registry cohort**,  N = 114,021*^1^* |
| --- | --- | --- |
| **Age (y)** | 0 (0%) | 0 (0%) |
| **Gender** | 1 (<0.1%) | 3 (<0.1%) |
| **Body Mass Index (kg/m²)** | 248 (7.2%) | 4,142 (3.6%) |
| **At least one comorbidity*** | 0 (0%) | 0 (0%) |
| **Admission Type** | 0 (0%) | 1,560 (1.4%) |
| **Admission Source** | 0 (0%) | 395 (0.3%) |
| **Planned Admission** | 0 (0%) | 0 (0%) |
| **Hospital Type** | 0 (0%) | 0 (0%) |
| **APACHE III Score** | 23 (0.7%) | 1,531 (1.3%) |
| **APACHE IV Mortality Probability** | 23 (0.7%) | 1,531 (1.3%) |
| **APACHE IV Risk Category** | 23 (0.7%) | 1,531 (1.3%) |
| **Mechanical Ventilation (first 24h)** | 0 (0%) | 0 (0%) |
| **Cardiothoracic Surgical** | 0 (0%) | 1 (<0.1%) |
| **Community Acquired Pneumonia** | 1 (<0.1%) | 2 (<0.1%) |
| **Sepsis** | 0 (0%) | 0 (0%) |
| **Out-of-Hospital Cardiac Arrest** | 0 (0%) | 46 (<0.1%) |
| **Trauma** | 0 (0%) | 0 (0%) |
| **Stroke** | 0 (0%) | 0 (0%) |
| **LOS ICU (days)** | 0 (0%) | 0 (0%) |
| **LOS Hospital (days)** | 0 (0%) | 819 (0.7%) |
| **Hospital Mortality** | 0 (0%) | 0 (0%) |
| **3-Month Mortality** | 0 (0%) | 0 (0%) |
| **6-Month Mortality** | 0 (0%) | 0 (0%) |
| **12-Month Mortality** | 0 (0%) | 0 (0%) |
| *^1^*Frequency (%) | | |
| *Immunological Insufficiency, Renal Insufficiency, Metastasized Neoplasm, Respiratory Insufficiency, Cardiovascular Insufficiency, Hematological Malignancy, or Liver Cirrhosis. | | |
| APACHE = Acute Physiology and Chronic Health Evaluation, LOS = Length Of Stay, ICU = Intensive Care Unit, IQR = Inter-Quartile Range, SD = Standard Deviation. | | |

Supplemental Table 2. Differences in demographic, clinical and ICU admission characteristics between ICU survivors in the PROM-based cohort and in the national registry cohort, admitted in 2019 or 2022: a subgroup analysis on cardiothoracic surgery patients.

| **Variable** | **PROM-based cohort**,  N = 978*^1^* | **National registry cohort**,  N = 24,867*^1^* | **SMD** |
| --- | --- | --- | --- |
| **Age (y)** | 65 (±10) | 66 (±11) | -0.10 |
| **Gender** |  |  | 0.02 |
| Female | 249 (25%) | 6,565 (26%) |  |
| Male | 729 (75%) | 18,302 (74%) |  |
| **Body Mass Index (kg/m²)** | 27.2 (±4.3) | 27.4 (±4.6) | -0.05 |
| **At least one comorbidity*** | 142 (15%) | 3,533 (14%) | 0.01 |
|  |  |  |  |
| **Admission Type** |  |  | 0.09 |
| Medical | 0 (0%) | 0 (0%) |  |
| Emergency Surgical | 47 (4.8%) | 1,636 (6.9%) |  |
| Planned Surgical | 931 (95%) | 22,150 (93%) |  |
| **Admission Source** |  |  | 0.32 |
| Operating Room | 947 (97%) | 22,496 (91%) |  |
| Emergency Room | 0 (0%) | 32 (0.1%) |  |
| Nursing Ward | 13 (1.3%) | 1,731 (7.0%) |  |
| ICU/CCU/Rec/Spec/MCU** | 2 (0.2%) | 297 (1.2%) |  |
| Other | 16 (1.6%) | 290 (1.2%) |  |
| **Planned Admission** | 935 (96%) | 22,767 (92%) | 0.17 |
| **Hospital Type** |  |  | 1.6 |
| Academic | 975 (100%) | 10,733 (43%) |  |
| Teaching | 3 (0.3%) | 11,846 (48%) |  |
| General | 0 (0%) | 2,288 (9.2%) |  |
| **APACHE III Score** | 54 (±16) | 46 (±16) | 0.51 |
| **APACHE IV** |  |  |  |
| **Mortality Probability** | 0.04 (±0.06) | 0.03 (±0.05) | 0.20 |
| **Risk Category** |  |  | 0.05 |
| Low | 965 (99%) | 24,651 (99%) |  |
| Medium | 12 (1.2%) | 198 (0.8%) |  |
| High | 0 (0%) | 13 (<0.1%) |  |
| **Mechanical Ventilation (first 24h)** | 937 (96%) | 22,933 (92%) | 0.15 |
| **Primary Admission Diagnosis** |  |  |  |
| **Community Acquired Pneumonia** | 0 (0%) | 0 (0%) | 0.00 |
| **Sepsis** | 1 (0.1%) | 75 (0.3%) | 0.04 |
| **Out-of-Hospital Cardiac Arrest** | 0 (0%) | 3 (<0.1%) | 0.02 |
| **Trauma** | 0 (0%) | 6 (<0.1%) | 0.02 |
| **Stroke** | 0 (0%) | 0 (0%) | 0.00 |
|  |  |  |  |
| **LOS (days)** |  |  |  |
| **ICU** | 1.8 (±2.6) | 1.8 (±3.7) | 0.01 |
| **Hospital** | 10 (±8) | 10 (±10) | 0.00 |
| **Mortality** |  |  |  |
| **Hospital** | 5 (0.5%) | 189 (0.8%) | 0.03 |
| **3-Month** | 9 (0.9%) | 452 (1.8%) | 0.08 |
| **6-Month** | 13 (1.3%) | 594 (2.4%) | 0.08 |
| **12-Month** | 17 (1.7%) | 832 (3.3%) | 0.10 |
| *^1^*Mean (±SD) or Frequency (%) | | | |
| *Immunological Insufficiency, Renal Insufficiency, Metastasized Neoplasm, Respiratory Insufficiency, Cardiovascular Insufficiency, Hematological Malignancy, or Liver Cirrhosis. | | | |
| **Intensive Care Unit, Coronary Care Unit, Recovery, Special Care Unit, or Medium Care Unit. | | | |
| APACHE = Acute Physiology and Chronic Health Evaluation, LOS = Length Of Stay, ICU = Intensive Care Unit, SMD = Standardized Mean Difference, SD = Standard Deviation. | | | |

Supplemental Table 3. Differences in demographic, clinical and ICU admission characteristics between ICU survivors in the PROM-based cohort and in the national registry cohort, admitted in 2019 or 2022: a subgroup analysis on non-cardiothoracic surgery patients in the six MONITOR-IC hospitals.

| **Variable** | **PROM-based cohort**,  N = 2,454*^1^* | **National registry cohort**,  N = 4,659*^1^* | **SMD** |
| --- | --- | --- | --- |
| **Age (y)** | 62 (±15) | 59 (±18) | 0.18 |
| **Gender** |  |  | 0.06 |
| Female | 984 (40%) | 2,011 (43%) |  |
| Male | 1,469 (60%) | 2,645 (57%) |  |
| **Body Mass Index (kg/m²)** | 27.1 (±5.7) | 26.4 (±6.0) | 0.11 |
| **At least one comorbidity*** | 710 (29%) | 1,265 (27%) | 0.04 |
|  |  |  |  |
| **Admission Type** |  |  | 0.33 |
| Medical | 1,474 (60%) | 3,500 (75%) |  |
| Emergency Surgical | 404 (16%) | 541 (12%) |  |
| Planned Surgical | 576 (23%) | 617 (13%) |  |
| **Admission Source** |  |  | 0.39 |
| Operating Room | 781 (32%) | 810 (17%) |  |
| Emergency Room | 843 (34%) | 2,225 (48%) |  |
| Nursing Ward | 595 (24%) | 1,012 (22%) |  |
| ICU/CCU/Rec/Spec/MCU** | 52 (2.1%) | 99 (2.1%) |  |
| Other | 183 (7.5%) | 504 (11%) |  |
| **Planned Admission** | 524 (21%) | 563 (12%) | 0.25 |
| **Hospital Type** |  |  | 0.35 |
| Academic | 970 (40%) | 1,147 (25%) |  |
| Teaching | 1,263 (51%) | 2,784 (60%) |  |
| General | 221 (9.0%) | 728 (16%) |  |
| **APACHE III Score** | 59 (±25) | 56 (±25) | 0.13 |
| **APACHE IV** |  |  |  |
| **Mortality Probability** | 0.19 (±0.20) | 0.17 (±0.19) | 0.09 |
| **Risk Category** |  |  | 0.08 |
| Low | 1,949 (80%) | 3,788 (81%) |  |
| Medium | 370 (15%) | 714 (15%) |  |
| High | 113 (4.6%) | 147 (3.2%) |  |
| **Mechanical Ventilation (first 24h)** | 1,204 (49%) | 1,786 (38%) | 0.22 |
| **Primary Admission Diagnosis** |  |  |  |
| **Community Acquired Pneumonia** | 178 (7.3%) | 321 (6.9%) | 0.01 |
| **Sepsis** | 209 (8.5%) | 346 (7.4%) | 0.04 |
| **Out-of-Hospital Cardiac Arrest** | 125 (5.1%) | 172 (3.7%) | 0.07 |
| **Trauma** | 203 (8.3%) | 349 (7.5%) | 0.03 |
| **Stroke** | 50 (2.0%) | 155 (3.3%) | 0.08 |
|  |  |  |  |
| **LOS (days)** |  |  |  |
| **ICU** | 4.7 (±7.8) | 3.8 (±8.1) | 0.11 |
| **Hospital** | 16 (±16) | 13 (±16) | 0.19 |
| **Mortality** |  |  |  |
| **Hospital** | 35 (1.4%) | 231 (5.0%) | 0.20 |
| **3-Month** | 103 (4.2%) | 559 (12%) | 0.29 |
| **6-Month** | 153 (6.2%) | 705 (15%) | 0.29 |
| **12-Month** | 226 (9.2%) | 911 (20%) | 0.30 |
| *^1^*Mean (±SD) or Frequency (%) | | | |
| *Immunological Insufficiency, Renal Insufficiency, Metastasized Neoplasm, Respiratory Insufficiency, Cardiovascular Insufficiency, Hematological Malignancy, or Liver Cirrhosis. | | | |
| **Intensive Care Unit, Coronary Care Unit, Recovery, Special Care Unit, or Medium Care Unit. | | | |
| APACHE = Acute Physiology and Chronic Health Evaluation, LOS = Length Of Stay, ICU = Intensive Care Unit, SMD = Standardized Mean Difference, SD = Standard Deviation. | | | |

Supplemental Table 4. Differences in demographic, clinical and ICU admission characteristics between ICU survivors in the PROM-based cohort and in the national registry cohort, admitted in 2019 or 2022: a subgroup analysis on non-cardiothoracic surgery, academic hospital patients.

| **Variable** | **PROM-based cohort**,  N = 970*^1^* | **National registry cohort**,  N = 17,523*^1^* | **SMD** |
| --- | --- | --- | --- |
| **Age (y)** | 59 (±16) | 57 (±17) | 0.09 |
| **Gender** |  |  | 0.02 |
| Female | 383 (40%) | 6,786 (39%) |  |
| Male | 586 (60%) | 10,734 (61%) |  |
| **Body Mass Index (kg/m²)** | 26.4 (±5.0) | 26.5 (±5.8) | -0.02 |
| **At least one comorbidity*** | 325 (34%) | 5,647 (32%) | 0.03 |
|  |  |  |  |
| **Admission Type** |  |  | 0.46 |
| Medical | 409 (42%) | 10,625 (61%) |  |
| Emergency Surgical | 199 (21%) | 3,762 (21%) |  |
| Planned Surgical | 362 (37%) | 3,124 (18%) |  |
| **Admission Source** |  |  | 0.38 |
| Operating Room | 496 (51%) | 5,835 (33%) |  |
| Emergency Room | 211 (22%) | 5,853 (33%) |  |
| Nursing Ward | 123 (13%) | 2,908 (17%) |  |
| ICU/CCU/Rec/Spec/MCU** | 29 (3.0%) | 552 (3.2%) |  |
| Other | 111 (11%) | 2,361 (13%) |  |
| **Planned Admission** | 379 (39%) | 2,978 (17%) | 0.51 |
| **APACHE III Score** | 56 (±23) | 61 (±26) | -0.19 |
| **APACHE IV** |  |  |  |
| **Mortality Probability** | 0.17 (±0.19) | 0.21 (±0.22) | -0.21 |
| **Risk Category** |  |  | 0.21 |
| Low | 789 (82%) | 12,610 (74%) |  |
| Medium | 138 (14%) | 3,580 (21%) |  |
| High | 32 (3.3%) | 891 (5.2%) |  |
| **Mechanical Ventilation (first 24h)** | 585 (60%) | 10,113 (58%) | 0.05 |
| **Primary Admission Diagnosis** |  |  |  |
| **Community Acquired Pneumonia** | 14 (1.4%) | 726 (4.1%) | 0.16 |
| **Sepsis** | 33 (3.4%) | 1,174 (6.7%) | 0.15 |
| **Out-of-Hospital Cardiac Arrest** | 30 (3.1%) | 946 (5.4%) | 0.11 |
| **Trauma** | 141 (15%) | 2,229 (13%) | 0.05 |
| **Stroke** | 32 (3.3%) | 845 (4.8%) | 0.08 |
|  |  |  |  |
| **LOS ICU (days)** |  |  |  |
| **ICU** | 4.8 (±9.1) | 4.8 (±8.9) | 0.01 |
| **Hospital** | 17 (±18) | 19 (±22) | -0.09 |
| **Mortality** |  |  |  |
| **Hospital** | 12 (1.2%) | 1,005 (5.7%) | 0.25 |
| **3-Month** | 42 (4.3%) | 2,147 (12%) | 0.29 |
| **6-Month** | 61 (6.3%) | 2,716 (15%) | 0.30 |
| **12-Month** | 90 (9.3%) | 3,476 (20%) | 0.30 |
| *^1^*Mean (±SD) or Frequency (%) | | | |
| *Immunological Insufficiency, Renal Insufficiency, Metastasized Neoplasm, Respiratory Insufficiency, Cardiovascular Insufficiency, Hematological Malignancy, or Liver Cirrhosis. | | | |
| **Intensive Care Unit, Coronary Care Unit, Recovery, Special Care Unit, or Medium Care Unit. | | | |
| APACHE = Acute Physiology and Chronic Health Evaluation, LOS = Length Of Stay, ICU = Intensive Care Unit, SMD = Standardized Mean Difference, SD = Standard Deviation. | | | |

Supplemental Table 5. Differences in demographic, clinical and ICU admission characteristics between ICU survivors in the PROM-based cohort and in the national registry cohort, admitted in 2019 or 2022: a subgroup analysis on non-cardiothoracic surgery, non-academic hospital patients.

| **Variable** | **PROM-based cohort**,  N = 1,484*^1^* | **National registry cohort**,  N = 71,631*^1^* | **SMD** |
| --- | --- | --- | --- |
| **Age (y)** | 64 (±14) | 62 (±16) | 0.10 |
| **Gender** |  |  | 0.05 |
| Female | 601 (40%) | 30,753 (43%) |  |
| Male | 883 (60%) | 40,878 (57%) |  |
| **Body Mass Index (kg/m²)** | 27.5 (±6.0) | 27.0 (±6.3) | 0.08 |
| **At least one comorbidity*** | 385 (26%) | 17,170 (24%) | 0.05 |
|  |  |  |  |
| **Admission Type** |  |  | 0.30 |
| Medical | 1,065 (72%) | 45,096 (63%) |  |
| Emergency Surgical | 205 (14%) | 7,485 (11%) |  |
| Planned Surgical | 214 (14%) | 18,583 (26%) |  |
| **Admission Source** |  |  | 0.33 |
| Operating Room | 285 (19%) | 22,347 (31%) |  |
| Emergency Room | 632 (43%) | 25,458 (36%) |  |
| Nursing Ward | 472 (32%) | 17,048 (24%) |  |
| ICU/CCU/Rec/Spec/MCU** | 23 (1.5%) | 1,010 (1.4%) |  |
| Other | 72 (4.9%) | 5,408 (7.6%) |  |
| **Planned Admission** | 145 (9.8%) | 15,895 (22%) | 0.34 |
| **APACHE III Score** | 61 (±26) | 54 (±25) | 0.28 |
| **APACHE IV** |  |  |  |
| **Mortality Probability** | 0.20 (±0.21) | 0.15 (±0.18) | 0.24 |
| **Risk Category** |  |  | 0.18 |
| Low | 1,160 (79%) | 59,774 (85%) |  |
| Medium | 232 (16%) | 8,855 (13%) |  |
| High | 81 (5.5%) | 1,918 (2.7%) |  |
| **Mechanical Ventilation (first 24h)** | 619 (42%) | 20,517 (29%) | 0.28 |
| **Primary Admission Diagnosis** |  |  |  |
| **Community Acquired Pneumonia** | 164 (11%) | 4,479 (6.3%) | 0.17 |
| **Sepsis** | 176 (12%) | 5,260 (7.3%) | 0.15 |
| **Out-of-Hospital Cardiac Arrest** | 95 (6.4%) | 1,783 (2.5%) | 0.19 |
| **Trauma** | 62 (4.2%) | 4,312 (6.0%) | 0.08 |
| **Stroke** | 18 (1.2%) | 2,156 (3.0%) | 0.13 |
|  |  |  |  |
| **LOS (days)** |  |  |  |
| **ICU** | 4.6 (±6.8) | 3.3 (±6.6) | 0.20 |
| **Hospital** | 14 (±14) | 12 (±14) | 0.15 |
| **Mortality** |  |  |  |
| **Hospital** | 23 (1.5%) | 3,131 (4.4%) | 0.17 |
| **3-Month** | 61 (4.1%) | 6,892 (9.6%) | 0.22 |
| **6-Month** | 92 (6.2%) | 8,861 (12%) | 0.21 |
| **12-Month** | 136 (9.2%) | 11,767 (16%) | 0.22 |
| *^1^*Mean (±SD) or Frequency (%) | | | |
| *Immunological Insufficiency, Renal Insufficiency, Metastasized Neoplasm, Respiratory Insufficiency, Cardiovascular Insufficiency, Hematological Malignancy, or Liver Cirrhosis. | | | |
| **Intensive Care Unit, Coronary Care Unit, Recovery, Special Care Unit, or Medium Care Unit. | | | |
| APACHE = Acute Physiology and Chronic Health Evaluation, LOS = Length Of Stay, ICU = Intensive Care Unit, SMD = Standardized Mean Difference, SD = Standard Deviation. | | | |
